# Inferring Causalities of Environmental and Genetic Factors for Differential Somatic Cell Count and Mastitis Pathogens in Dairy Cows Using Structural Equation Modelling

**DOI:** 10.3390/genes14112102

**Published:** 2023-11-19

**Authors:** Patricia Wagner, Kerstin Brügemann, Tong Yin, Petra Engel, Sven König

**Affiliations:** Institute of Animal Breeding and Genetics, Justus-Liebig-University of Gießen, Ludwigstr. 21b, 35390 Giessen, Germany

**Keywords:** dairy cow, udder heath, structural equation model, compost-bedded pack barns, differential somatic cell count, specific mastitis pathogens, genomic data

## Abstract

The aim of this study was to establish and evaluate a structural equation model to infer causal relationships among environmental and genetic factors on udder health. For this purpose, 537 Holstein Friesian cows were genotyped, and milk samples were analyzed for novel traits including differential somatic cell counts and specific mastitis pathogens. In the structural model, four latent variables (intramammary infection (IMI), production, time and genetics) were defined, which were explained using manifest measurable variables. The measurable variables included udder pathogens and somatic differential cell counts, milk composition, as well as significant SNP markers from previous genome-wide associations for major and minor pathogens. The housing system effect (i.e., compost-bedded pack barns versus cubicle barns) indicated a small influence on IMI with a path coefficient of −0.05. However, housing system significantly affected production (0.37), with ongoing causal effects on IMI (0.17). Thus, indirect associations between housing and udder health could be inferred via structural equation modeling. Furthermore, genotype by environment interactions on IMI can be represented, i.e., the detection of specific latent variables such as significant SNP markers only for specific housing systems. For the latent variable genetics, especially one SNP is of primary interest. This SNP is located in the *EVA1A* gene, which plays a fundamental role in the MAPK1 signaling pathway. Other identified genes (e.g., *CTNNA3* and *CHL1*) support results from previous studies, and this gene also contributes to mechanisms of the MAPK1 signaling pathway.

## 1. Introduction

Diseases of the udder are considered as one of the most important clinical infections in dairy cows with strong detrimental effects on farm economy [1]. However, in terms of underlying genetic and physiological mechanisms and with regard to pathogenesis, udder infections are very complex and depend on a variety of factors, including milk yield, lactation stage, genetics, type of pathogens, and also on farm-specific characteristics [2,3]. Farm characteristics address alternative animal friendly housing systems appreciated by the society, such as compost-bedded pack barns. From an animal perspective, compost-bedded pack barns improve animal welfare, animal health and longevity [4]. On the other hand, due to the mixture of the substrate of bedding materials and manure, there may be an increased risk of bacterial infections in the udder [5].

As pointed out in some publications [6], the cow milk quality requirements as defined by the European Union are based on herd averages for somatic cell count (SCC) with a maximum of 400,000 cells/mL and a bacterial standard plate count. However, the type of pathogens can greatly vary. The type of pathogens not only determine whether an intramammary infection induces an acute or a subclinical mastitis but also has specific effects on the overall immune system. Specific defense mechanisms might be activated, thus affecting the somatic cell count in milk, which is the standard parameter for indicating udder health status [7]. However, the composition of the somatic cells might also vary depending on the type of infection, suggesting differential somatic cell count analyses. Furthermore, genetic cascades are triggered by the immune system, which can vary individually. Intramammary infection impairs milk yield and milk composition. Additionally, in recursive biological systems, the level of milk yield and composition might influence the susceptibility of an udder infection [8,9]. In addition, from an “environmental” perspective, the age of the animal, the status of nutrition and many other factors such as climate play a role in determining an animal’s susceptibility for an infection [2,3].

In order to fully understand and resolve such complex and various influencing factors and their interrelationships, alternative modeling approaches are needed. Regression analyses are used as a common tool to explore one-way relationships by neglecting possible recursive or mutual associations [10]. Structural equation models (SEM), on the other hand, allow for flexible and comprehensive approaches to examine the relationships between variables in a hypothetical model [11]. Extended SEMs depict associations among measurable parameters (manifest variables), and additionally enable the estimation of parameters that cannot be measured or recorded directly among themselves, the so-called underlying latent variables [12]. De los Campos et al. [13] and Wu et al. [14] have already used SEM to infer relationships between udder health (via somatic cell count) and milk yield both phenotypically and genetically, but in their modeling approach, they ignored mutual associations among environmental effects. Another advantage of an SEM is that both direct and indirect effects can be modeled simultaneously, also in a recursive framework [15]. Detilleux et al. used SEM to obtain a basic understanding of the many different factors involved in clinical mastitis in the risk for infections and tolerance mechanisms [3,16]. The relationships among the latent variables, among the manifest variables as well as among the latent and manifest, are denoted as loading coefficients or path coefficients.

The objective of the present study was to apply SEM to infer causal relationships on IMI based on a detailed recording for differential somatic cell counts and specific mastitis pathogens as well as specifically selected SNP markers. In this regard, a two-step strategy was applied; first, a genome-wide association study (GWAS) was used to detect significant SNPs, and afterwards, in step 2, these SNPs were analyzed using enhanced SEM approaches. 

## 2. Materials and Methods

### 2.1. Animal Ethics Statement

Data considered in the present study are based on milk samples from routine milk recording and genotypes used for the official national genetic evaluations. No extra animal experiments were conducted. Thus, following the guidelines of the German animal welfare legislation, a specific ethical approval was not required.

### 2.2. Farms, Animals and Sampling

Cow milk samples were collected from the individual udders of six Holstein dairy cattle herds located in the German federal states of Hesse and North Rhine-Westphalia for all ongoing studies. The herds for this project were selected based on the criteria for selecting case (i.e., herds with the compost farming system) and control herds (i.e., conventional cubicle barns) as defined in the collaborative EU FreeWalk project [17]. The two housing systems compost-bedded pack barns and cubicle barns were identical with regard to herd size, production level, location, climatic conditions, milking system, feeding and breeding aspects in order to ensure an objective comparison. The only, and a large, difference was the housing system; i.e., compost-bedded pack barns in contrast to conventional cubicle barns. Overall, three farms represented the compost system and three other farms had the cubicle system, while two farms had both the systems. Allocation of cows to the different sub-herds in the farms with both systems is described in our previous study by Wagner et al. [18]. The current study considered 587 first and second parity Holstein Friesian dairy cows. A large fraction of all cows, 79%, was in first lactation. A total of 44% of all cows were kept in compost farms, and 56% of all cows were in the conventional cubicle farms, indicating a very similar data distribution across both the housing systems.

For ongoing laboratory milk analyses, we considered milk samples from 2198 udder quarters of these 587 cows. The milk analyses for specific pathogens in the Landesbetrieb Hessen followed the DVG guidelines [19]. The specific mastitis pathogens were classified into major pathogens (MAJOR) or minor pathogens (MINOR). The classification was conducted according to the severity of an infection and immune responses, and does not indicate any prevalence. The same classification of mastitis pathogens according to the infection status was considered in several previous udder health studies, especially when inferring genetic and physiological mechanisms [20,21,22,23]. Accordingly, the category MAJOR included the pathogens *Aerococcus* sp., *Aesculin hydrolyzing streptococci*, *Candida krusei*, *Enterococcus* sp., *Escherichia coli*, *Lactococcus* sp., *Staphylococcus aureus*, *Streptococcus dysgalactiae*, *Streptococcus uberis*, mold fungus and *Proteus* sp. The category MINOR included *Coagulase-negative staphylococci* and *Corynebacterium* sp. One udder quarter of at least one pathogen within the defined groups, MAJOR and MINOR, resulted in score = 1 for the respective total group; otherwise, score = 0 was assigned. At cow level, the score = 1 was assigned for the total group (MAJOR, MINOR) if the respective pathogen was detected in at least one udder quarter; otherwise, score = 0 was applied.

Differential cells were counted under the microscope in our own milk analysis laboratory at Justus-Liebig-University, Giessen. Differential cell counting included the cell fractions for lymphocytes, macrophages and polymorphonuclear leucocytes (PMN) in a 50 mL milk sample per udder quarter following official guidelines and protocols [24,25]. Milk samples with a small number of counted cells (<30 counted cells per sample) were excluded from the ongoing modeling approaches. The sum of all determined lymphocytes, macrophages and PMN was defined as 100%, and the respective percentages of the specific cell types were used as variables in the SEM. The descriptive statistics of the udder health traits are shown in Table 1.

### 2.3. Genome-Wide Associations

Only the significant SNPs from GWAS were included in the ongoing SEM. Genome-wide associations were performed for SNP main effects and SNP x housing system interaction effects. A significant interaction means that the SNP significantly affects udder health in compost-bedded pack barns, but not in cubicle barns. In this regard, we followed SNP data preparation and the statistical methods as outlined in our previous study by Wagner et al. [6]. For this, 277 Holstein Friesian (HF) cows were genotyped with the *Illumina BovineSNP50 Bead Chip V2.* An additional dataset including 273 first parity HF cows was genotyped with the *Illumina BovineSNP50 Bead Chip V3*. After quality control of the SNP data via the software package PLINK, version 1.9 [26], 43,095 SNPs from 550 genotyped cows were available for the ongoing genomic studies. Criteria for SNP quality control implied consideration of Bos Taurus autosomes, the exclusion of SNPs with a minor allele frequency lower than 0.01, the exclusion of SNPs with a call rate lower than 0.90 and the exclusion of SNPs significantly deviating (*p* < 0.001) from the Hardy–Weinberg equilibrium. ARS1.2 assembly [27] was used for remapping the positions of the SNP markers.

The algorithm for the estimation of SNP effects and significances is incorporated into our own R package, named GWAInter.R, which can be downloaded at https://jlupub.ub.uni-giessen.de/ (accessed on 20 April 2023). The respective statistical model 1 in matrix notation for the GWAS was defined as follows:

y = Xb + x_snpi_ b_snpi_ + x_interi_ b_interi_ + Zg + e (1)

where y = a vector of observations for the pathogens MAJOR or MINOR (cow level) in consecutive runs; b = a vector of fixed effects including the herd test day, the housing system cubicle barn or compost-bedded pack barn, parity, and the person from the milk laboratory analyzing the milk samples; X = incidence matrix for fixed effects; x_snpi_ = a vector of SNP genotypes; b_snpi_ = a regression coefficient for the main effect of the i*th* SNP marker; x_interi_ = a vector of genotypes for cows kept in compost-bedded pack barns; b_interi_ = a regression coefficient for the SNP x housing system interaction effect of the i*th*-SNP marker; g = a vector of random additive-genetic effects following N(0, Gσ^2^_g_); G = the genomic relationship matrix which was constructed as defined by Yang et al. [28] by excluding the respective candidate SNP; σ^2^_g_ = additive-genetic variance; Z = incidence matrix for the random additive-genetic effects; e = vector for the random residual effects following N(0, Iσ^2^_e_), I = identity matrix; σ^2^_e_ = residual variance.

The estimation for the required additive-genetic and residual variances for MAJOR and MINOR was performed using the model y = Xb + Zg + e, with the effects as stated above for model 1 and applying the software package “gaston” [29]. Our software package GWAInter.R version 1.0 utilizes generalized least squares approach as outlined by Halli et al. [30] for the estimation of the SNP main and interaction effects. In this regard, we used a Wald-test statistic [31]. The respective chi^2^ value for the main and interaction effects is the ratio of the respective squared regression coefficient divided by the variance of the regression coefficients at 1 degree of freedom. Significance thresholds were defined based on the strict Bonferroni correction with *P_Bonf_* = 0.05/no. of SNPs and the more relaxed suggestive threshold with *P_sugg_* = 0.05/number of independent SNPs. The number of SNPs was 43,095, and the number of independent SNPs was 4479. The number of independent SNPs was calculated considering linkage disequilibrium ≤0.15 in chromosomal segments with 500 markers.

The last step was the annotation of potential candidate gens. The definition of a candidate gene implied at least one significantly associated SNP, which is directly located in the gene or located in a surrounding segment 200 kb up- and downstream. For gene annotations, we used the databases ENSEMBL and NCBI [32,33]. For the interpretation of gene functions and related physiological pathways with focus on the modelings in the ongoing SEM, we referred to the Kyoto Encyclopedia of Genes and Genomes and the NCBI database [33,34].

A total of 41 SNPs were significant for both SNP main and SNP interaction effects. Based on SEM evaluations for goodness of fit criteria (see Section 3.1), 13 SNPs from both categories, MAJOR and MINOR, were integrated into the final SEM. These significant SNPs are listed in Table 2 along with respective candidate gene information and location.

### 2.4. Structural Equation Model

The package lavaan (version 0.6–9) in R (version 3.6.2) [35] was used for the development and application of the SEM [36]. The SEM is composed of a structural model, several reflective measurement models and a formative measurement model. The general SEM (Model (2)) was

η = B⋅η + Γ ⋅ ξ + ζ (structural equation model)(2)

The formative measurement Models (3) and (4) were

x = Λx ⋅ ξ + δ (Measurement model for latent exogenous variables ξ)(3)

y = Λy ⋅ η + ε (Measurement model for latent endogenous variables η)(4)

The reflective measurement Models (5) and (6) were

ξ = x ⋅ Пξ + δξ (Measurement model for latent exogenous variables ξ)(5)

η = y ⋅ Пη + δη (Measurement model for latent endogenous variables η)(6)

where η = a vector representing endogenous latent variables (η_1_ = production, η_2_ = intramammary infection und η_3_ = genetics); ξ = a vector of exogenous latent variables (ξ_1_ = time); ζ = indicating the residual variable, since endogenous latent variables are not completely explained by the exogenous latent variables, and the complete impact for factors that were not considered in the model. The coefficient matrices B and Γ show the interdependence relationships B between latent endogenous variables and Γ between latent endogenous and exogenous variables); x and y = vectors representing the indicator variables, as described in Table 3; Λx and Λy = vectors of the path or loading coefficients (λn); П_ξ_ and П_η_ = vectors of multiple regression or weighting coefficients between an (endogenous or exogenous) latent variable and the assigned indicator variables; δ or ε = vectors of the exogenous or endogenous residuals.

## 3. Results

### 3.1. Overall Structural Equation Model Evaluation

The overall goodness fit of the model was assessed by applying a χ^2^ goodness of fit test and alternative fit indices. Such applications induce the standardized root-mean-square residual (0.101) (SRMR), the root-mean-square error of approximation (0.135) (RMSEA), the Tucker–Lewis index (0.244) (TLI) and the comparative fit index (0.312) (CFI). Based on these evaluation criteria, failed convergence status and unrealistic parameter estimates when including parity as indicator variable for the latent variable time, we decided to exclude parity as a cow-specific parameter from the SEM. This might be due to the extremely high proportion of first parity cows in our dataset and the strong auto-correlations between parity with other parameters including age at first calving and production traits.

**Figure 1:**
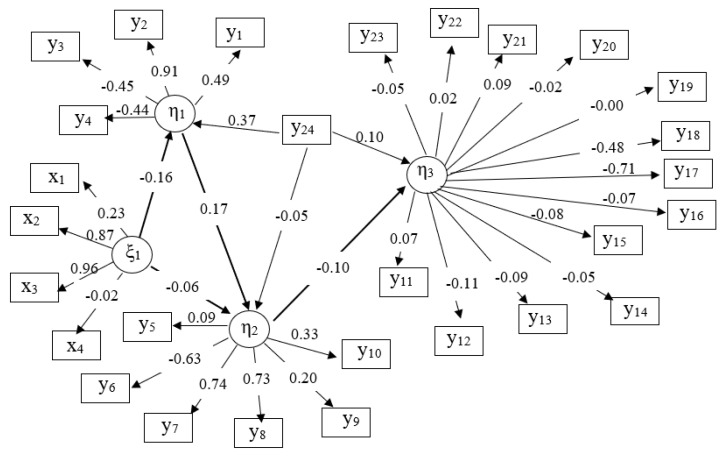
Path coefficients for causal relationships from the structural equation model with four latent variables (η_1_ = production, η_2_ = intramammary infection, η_3_ = genetics, ξ_1_ = time); x_1_ = barn age, x_2_ = average first calving age, x_3_ = average calving interval, x_4_ = lactation stage, y_1_ = fat content, y_2_ = protein content, y_3_ = lactose content, y_4_ = milk yield, y_5_ = average somatic cell count of the herd, y_6_ = lymphocytes, y_7_ = PMN, y_8_ = somatic cell count of test day, y_9_ = MAJOR, y_10_ = MINOR, y_11–23_ = significant SNPs from the previous GWAS as indicated in Table 2, y_24_ = housing system.

Completely standardized estimates of the parameters for the final SEM are shown in Figure 1. Four relationships were inferred among the latent variables in the SEM. In total, there are 28 measures (indicator or manifest variables) associated with latent variables enabling estimations of the respective latent variables. Information on the impact of latent constructs were obtained by assessing the path coefficients (λn). The possible range is from −1 to +1. A value of ≥0.2 or ≤−0.2 is generally considered as a relevant correlation [37].

### 3.2. Latent Variable Intramammary Infection

**Figure 2:**
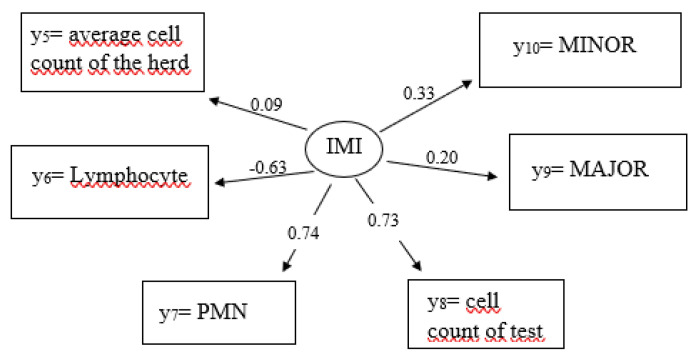
Path coefficients for causal relationships from the measurement model for the latent endogenous variable η_2_ = intramammary infection (IMI) and the manifest variables y_5_ = average somatic cell count of the herd, y_6_ = lymphocytes, y_7_ = PMN, y_8_ = individual somatic cell count of test day, y_9_ = MAJOR, y_10_ = MINOR system.

According to the measurement models, the overall average somatic cell count of the herd is a weak indicator (0.09) to explain an IMI (Figure 1 and Figure 2). In contrast, the influence of the individual somatic cow cell count from the test day explains a very accurate IMI (0.73). The path coefficients for lymphocytes and PMN (0.74) are also quite large (i.e., values close to −1 or close to 1), with a negative value for lymphocytes (−0.63). The path coefficients for the major and minor pathogens with 0.20 and 0.33, respectively, are slightly smaller than the estimates for the specific cell fractions.

### 3.3. Latent Variable Production

**Figure 3:**
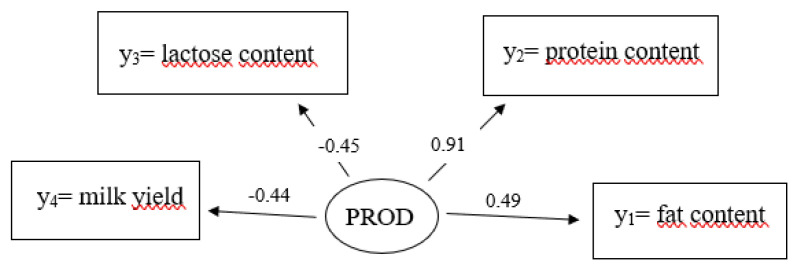
Path coefficients for causal relationships from the measurement model for the latent endogenous variable η_1_ = production (PROD) with the manifest variables y_1_ = fat content, y_2_ = protein content, y_3_ = lactose content, y_4_ = milk yield.

For the latent variable production (PROD), all four measurement variables indicate quite a large effect on production (Figure 1 and Figure 3). The highest path coefficient was found for protein content with 0.91, followed by fat content with 0.49. Lactose and milk yield are also important determinants with −0.45 and −0.44, respectively. 

### 3.4. Latent Variable Genetic

**Figure 4:**
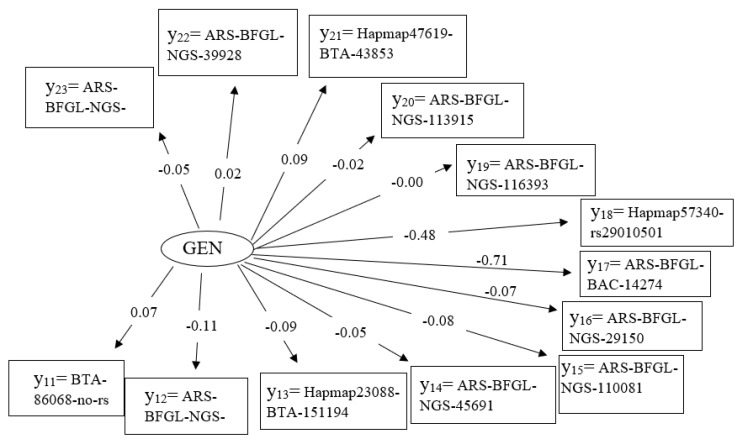
Path coefficients for causal relationships from the measurement model for the latent endogenous variable η_3_ = genetic (GEN) with the manifest variables y_11–23_ = significant SNP from the previous GWAS (Table 1).

The latent variable genetic (GEN) is determined by 13 measured variables, i.e., SNP effects. Two of these SNPs (y_17_ = ARS-BFGL-BAC-14274, y_18_ = Hapmap57340-rs29010501, both located on chromosome 11) indicate quite a strong effect, but the path coefficients of the remaining SNPs are close to zero (Figure 1 and Figure 4).

### 3.5. Latent Variable Time

**Figure 5:**
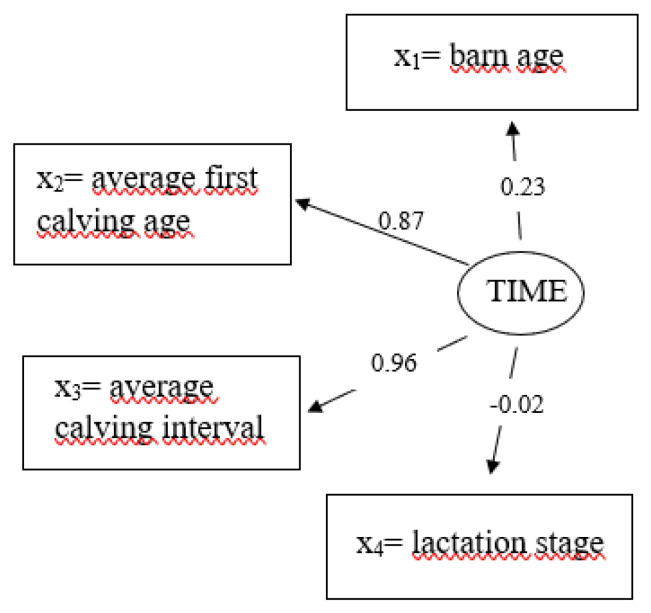
Path coefficients for causal relationships from the measurement model for the latent exogenous variable ξ_1_ = time (TIME) with the manifest variables x_1_ = barn age, x_2_ = average first calving age, x_3_ = average calving interval, x_4_ = lactation stage.

For the exogenous latent variable time (TIME), the path coefficients for average calving interval with 0.96 and the average calving age with 0.87 are quite large (Figure 5). A moderate effect was identified for barn age (0.23) and a negligible effect was identified for lactation stage (−0.02).

### 3.6. Relationships among Latent Variables

**Figure 6:**
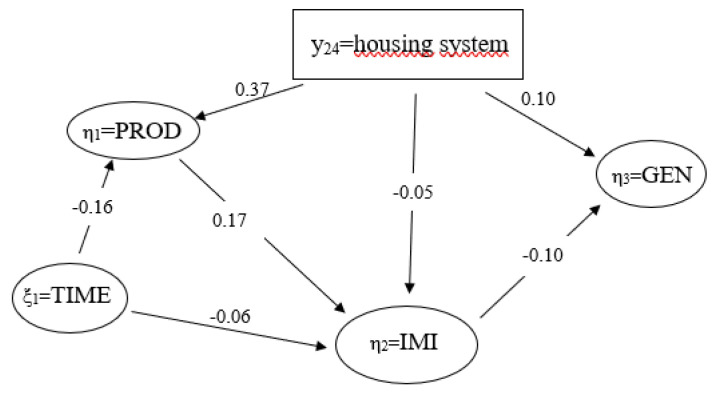
Path coefficients for causal relationships from the structural model with four latent variables (η_1_ = production, η_2_ = intramammary infection, η_3_ = genetics, ξ_1_ = time); y_24_ = housing system.

In the SEM framework, it is evident that the effect of the latent variable TIME on the latent variable IMI is quite small, with a path coefficient of −0.06 (Figure 6). The effect of TIME on the latent variable PROD was moderate (−0.16). Of similar magnitude was the effect of path coefficient of PROD on the latent variable IMI with 0.17, and the effect of the loading coefficient of the latent variable IMI on the latent variable GEN with −0.10.

In this model, the manifest variable housing system shows an effect on three latent variables. However, the respective path coefficient was quite small (0.05) for the latent variable IMI and for the latent variable GEN (0.10). The highest path coefficient from this model was 0.37, i.e., the effect of the measurement variable “housing system” on the latent variable PROD.

## 4. Discussion

### 4.1. Manifest Variables on Intramammary Infection and Production

In the present study, we used a holistic approach, which contributed to a deeper understanding of the mechanisms of udder health in dairy cattle in different housing systems simultaneously considering time, environmental effects and cow effects, combined with a variety of udder health indicators.

With regard to the overall measurement models, it is shown that the effect of the average herd somatic cell count is very small (0.09) to explain an intramammary infection. Accordingly, Beaudeau et al. [38] reported that herd cell count is a weak predictor for intramammary infections, implying the detailed recording of individual cell counts, preferably the generation of a longitudinal data structure by time. Especially, the effect of the individual somatic cell count at the nearest official test day on IMI was quite large (0.73), again supporting the results from the comprehensive udder health study by Beaudeau et al. [38]. The individual somatic cell count considerably changes in the course of an intramammary infection [39], and the large variation also explains the substantial SCC effects from the modeling approach. However, SCC as a single indicator is not sufficient to understand the mechanisms of udder health in detail. Riggio et al. [40] already showed strong associations between the increase in SCC and the status of an infection. Since the cell count and the cell composition depend on the type of pathogen [20], both lymphocytes and PMN, as well as major and minor were integrated into the SEM for the latent variable IMI. All four manifest variables had an effect on udder health and should be simultaneously considered to infer the physiological pathways of an intramammary infection [40].

The path coefficient for the specific cell fraction of the lymphocytes was negative (−0.63), since this cell fraction predominates, especially in the healthy udder quarter [41,42]. In contrast, the path coefficient of the PMN was positive (0.74). In the process of acute infections, the content of lymphocytes decreases, while the content of PMNs in the udder increases [18,43]. Hence, the results from the structural equation modeling approach support well-known physiological mechanisms. Both major and minor pathogens displayed moderate effects on the latent variable IMI. Interestingly, the influence of major pathogens (0.20) was smaller than the influence of minor pathogens (0.33). This could be due to the shift generally observed in the importance of mastitis pathogens, i.e., with a greater importance of minor than of major pathogens nowadays in the context of severe udder infections [18,44,45]. Accordingly, in the present study, more cows were significantly affected due to minor pathogens. Consequently, the results from the present SEM suggest the evaluation of alternative classifications of pathogens.

The four manifest variables to explain the latent variable PROD indicated a moderate to large effect in a range from −0.44 to 0.91. In this regard, the strongest effect with 0.91 was identified for protein content. Craig et al. [46] already showed that protein content is closely related to productivity. With increasing milk yield, the amount of protein decreases, explaining the opposite signs of the path coefficients of milk yield and protein content in our model [46]. Regarding udder health, protein content reacted very sensitively, especially to somatic cell count alterations, while fat content was more stable [9]. In our SEM analogy, path coefficients were larger for protein than for fat content. For milk quality traits, França et al. [9] indicated sensitivity of lactose contents to the udder health status, with pronounced contrast for infections with *Streptococcus* spp. or *Staphylococcus aureus*. Since our SEM is influenced by both types of pathogens and additional pathogens within the MAJOR and MINOR groups, this may depress individual path coefficients due to different inter-trait relationships of each pathogen, thereby explaining the smaller influence of lactose in our study.

### 4.2. Genetic Influence in the Structural Equation Model

For the latent variable GEN, 13 SNPs were included, but most of them only show a small influence in the SEM when additionally considering a large number of environmental characteristics. Nevertheless, these SNPs were significant for the major and minor pathogens in previous GWAS [6], but mostly located in chromosomal segments outside of functional genes. From a physiological perspective, the two groups, MAJOR and MINOR, contain many different species of pathogens that initiate very different immune responses, and thereby are modulated by many different genes [7]. For example, with regard to MAJOR, the immune response mechanisms of *Staphylococcus aureus* and *Escherichia coli* are different, triggering a cascade of specific genes for specific immune responses [47].

However, the SNP (ARS-BFGL-BAC-14274) of pathway y17 has a quite strong effect with −0.71. This SNP is located directly in the gene *EVA1A*. *EVA1A* is involved in autophagy and the programming of cell death [48,49]. In addition, this quite under-researched gene plays an important role by up- or down-regulating in the MAPK (mitogen-activated protein kinase) signaling pathway [28]. The MAPK pathway plays a fundamental role in udder health, with interactions of the *CHL1* gene [50,51]. However, direct consideration of *CHL1* in the SEM implied an only small path coefficient of y_11_ = 0.07. With regard to functional mechanisms and pathways of udder health, *CHL1* plays a role in the activation of the MAPK signaling pathway [50,51]. In a GWAS for the cell fraction PMN, ignoring housing system interaction effects, the potential candidate gene *CTNNA3* was identified, which also intervenes in the MAPK pathway [6]. In the case of clinical mastitis, MAPK signaling regulates inflammatory gene expression [52].

### 4.3. Overall Structural Equation Model Evaluations, Limitations and Prospects

In the SEM, the exogenous latent variable TIME contains four manifest variables. In this regard, the effect of lactation stage was quite small with a path coefficient of −0.02. In contrast, a stronger effect with 0.23 was identified for the age of the cow barn. This variable was integrated in the SEM to consider the experiences of management practices, especially in the context of the quite new compost farming system. Ivemeyer et al. [53] highlighted the significance of herd management on udder health and productivity, especially in alternative or novel housing systems. The effect of the average age was very strong at first calving (0.87) and the average calving interval (0.96). Accordingly, in standard mixed models, age at first calving significantly influenced milk yield and milk composition [54,55], as well as udder health [56]. A late age at first calving was associated with increased milk yield and an increased risk for udder infections [57], supported by the signs for the latent variables PROD and IMI in the present study. Drews et al. [58] assigned a separate latent variable to the two manifest variables age at first calving and calving interval in his SEM. The high path coefficient for the age at first calving with 0.98 reflect the estimate from the present study. In contrast, Detilleux et al. [3] considered average parity in the herd and the percentage of heifers in their modeling approach. However, when developing a SEM and assembling the manifest and latent variables, it is imperative to exclude similar variables with similar explanatory power or high collinearity. Otherwise, estimates from the SEM might be biased [59]. This was also a reason to exclude the variable parity from our finally applied SEM.

The SEM inferred causal relationships among a variety of udder health indicators and respective environmental and cow-associated factors. The direct housing system effect on udder health was quite small (−0.05). However, the housing system moderately affected the latent variable PROD (0.37), implying an indirect housing system effect on IMI through this pathway. Both the housing system and the latent variable IMI were moderately associated with the latent variable GEN. Hence, the SEM also indicates possible genotype by environment interactions, because of the specific reactions of udder health and immune response traits depending on the housing conditions. Overall, the present SEM revealed its potential to depict complex structures of udder health in dairy cows via the modeling of direct and indirect pathways. For the structural equation modeling, we considered the four latent variables IMI, PROD, TIME and GEN and integrated the variable housing system as a formative measurement model in this SEM. Furthermore, we modeled direct and indirect pathways for trait and effect relationships. However, such comprehensive analyses require a broad data structure based on different types of data, i.e., novel cow traits, cow-associated factors, housing characteristics, as well as “classical” environmental effects. Generation of such data structure implies tremendous efforts regarding labor, time and logistics. Consequently, in the present study, we neglected some additional possible environmental effects on an intramammary infection such as the climatic conditions in the barn. Gernand et al. [60] identified temperature and humidity close to the official test-day as major effects on clinical mastitis. However, overloading a SEM with more detailed environmental effects might lead to failed convergence, or to biased parameter estimates [61]. An alternative in this regard is to enlarge the cow trait dataset, but this is also a challenge for novel health traits as considered in the present study. Attempts to establish the so-called “cow training sets” for genomic selection, comprising innovative health traits for a large number of genotyped cows [62], might be the perfect database for ongoing and even more detailed SEM applications.

## 5. Conclusions

The applied SEM clearly inferred effects among response variables indicating udder health and environmental and cow-associated factors. Trait and modeling complexity was reduced by considering the latent variables. The direct effect of the housing system on the latent variable IMI was quite small, but the indirect pathway via PROD indicated housing system–IMI associations. For the latent variable GEN, especially one SNP is of primary interest. This SNP is located in the *EVA1A* gene, which plays a fundamental role in the MAPK1 signaling pathway. Other identified genes (e.g., *CTNNA3* and *CHL1*) support results from previous studies, and this gene also contributes to mechanisms of the MAPK1 signaling pathway. Overall, our SEM emphasizes the importance of this pathway for udder health in a very complex modeling context including a larger number of further environmental effects.

## Figures and Tables

**Table 1:** Descriptive statistics for the microscopic differential somatic cell counts and mastitis pathogens per udder quarter.

Udder Health Traits ^1^	Mean	Min	Max	SD
*Cell fractions (in relation to the* *total sum of all cell counts)* Macrophages	0.292	0.000	0.980	0.208
Lymphocytes	0.608	0.000	1.000	0.246
PMN	0.100	0.000	0.971	0.143
*Mastitis pathogens (in prevalences)* Negative samples	0.514	0.000	1.000	0.500
Minor pathogens	0.407	0.000	1.000	0.491
Major pathogens	0.030	0.000	1.000	0.171

^1^ Polymorphonuclear neutrophils (PMN), minor pathogens (including *Coagulase-negative staphylococci* and *Corynebacterium* sp.), major pathogens (including *Aerococcus* sp., *Aesculin hydrolyzing streptococci*, *Candida krusei*, *Enterococcus* sp., *Escherichia coli*, *Lactococcus* sp., *Staphylococcus aureus*, *Streptococcus dysgalactiae*, *Streptococcus uberis*, mold fungus and *Proteus* sp.).

**Table 2:** Genome-wide significances for SNP main effects (superscript M) and interaction effects (superscript I) with housing systems and annotated potential candidate genes for mastitis pathogens which were integrated into the SEM considering estimates and findings by Wagner et al. [6].

Trait	SNP	CHR	Position	*p*-Value SNP	SNP Located in a Gene	Gene Name
MAJOR	BTA-86068-no-rs (y = 11) ^M^	22	26048787	0.000000002563736 ^a^	yes	*CHL1*
	BTA-86068-no-rs (y = 11) ^I^	22	26048787	0.0000004836461 ^a^	yes	*CHL1*
	ARS-BFGL-NGS-39928 (y = 22) ^M^	26	38508625	0.000002509339 ^a^	-	-
	ARS-BFGL-BAC-14274 (y = 17) ^M^	11	44153677	0.000001737926 ^a^	yes	*EVA1A*
	Hapmap57340-rs29010501 (y = 18) ^M^	11	44928962	0.000003947047 ^b^	-	-
	Hapmap23088-BTA-151194 (y = 13) ^M^	1	152612216	0.000003844456 ^b^	yes	*HACL1*
	ARS-BFGL-NGS-60721 (y = 12) ^M^	1	35809354	0.000004774763 ^b^	-	-
	Hapmap47619-BTA-43853 (y = 21) ^M^	18	4489809	0.0000008244985 ^b^	-	-
	ARS-BFGL-NGS-110081 (y = 15) ^M^	4	41230144	0.000001551728 ^b^	-	-
	ARS-BFGL-NGS-45691 (y = 14) ^M^	2	127889562	0.00000764746 ^b^	-	-
	ARS-BFGL-NGS-29150 (y = 16) ^M^	5	108921269	0.000008442227 ^b^	-	-
	ARS-BFGL-NGS-116393 (y = 19) ^M^	11	104186003	0.000002801936 ^b^	yes	*ABO*
	ARS-BFGL-NGS-113915 (y = 20) ^M^	17	32550404	0.0000008220506 ^b^	-	-
MINOR	ARS-BFGL-NGS-112964 (y = 23) ^M^	14	68578807	0.000002708568 ^b^	-	-

MAJOR *Aerococcus* sp., *Aesculin hydrolyzing streptococci*, *Candida krusei*, *Enterococcus* sp., *Escherichia coli*, *Lactococcus* sp., *Staphylococcus aureus*, *Streptococcus dysgalactiae*, *Streptococcus uberis*, mold fungus and *Proteus* sp., MINOR *Coagulase-negative staphylococci* and *Corynebacterium* sp., ^a^ Bonferroni-corrected genome-wide significance and ^b^ less conservative threshold, y represent the vectors of the indicator variables of the structural equation model described in Table 2.

**Table 3:** Overview of the latent variables and their associated measurement variables and (if possible) their minimum and maximum values, mean values and standard deviation or their groups. Each of the latent variables are estimated by three or more measurement variables; η = the vector of endogenous latent variables; ξ = the vector of exogenous latent variables; ζ = the residual variable; x and y = the vectors of the indicator variables.

Latent Variables	Indicator Variables (Manifest Variables)	Range (Min–Max) or Groups	Mean	SD
η_1_ = Production (PROD)	y_1_ = fat content [in %]	2.43–7.6	4.88	0.73
	y_2_ = protein content [in %]	2.7–4.93	3.56	0.37
	y_3_ = lactose content [in %]	3.4–5.28	4.88	0.19
	y_4_ = milk yield [in kg]	<25, 25.1–30, 30.1–35, 35.1–40, >40	-	-
η_2_ = Intramammary infection (IMI)	y_5_ = average somatic cell count of the herd	5.05–5.58	5.30	0.14
	y_6_ = lymphocyte content [in %]	0–100	61.00	0.25
	y_7_ = PMN content [in %]	0–97	9.80	0.14
	y_8_ = somatic cell count of test day (the exact test day of our sampling)	2.64–11.16	2.35	2.09
	y_9_ = MAJOR	0, 1	-	-
	y_10_ = MINOR	0, 1	-	-
η_3_ = Genetic (GEN)	y_11_ − _23_ = significant SNP from GWAS			
ξ_1_ = Time (TIME)	x_1_ = barn age [in years]	1, 2, 3	-	-
	x_2_ = average first calving age [in days]	750–760, 760.1–775, 775.1–780, 780.1–800, >800	-	-
	x_3_ = average calving interval [in days]	382–432	407	0.17
	x_4_ = lactation stage [in days]	0–100, 100.1–200, 200.1–300, >300	-	-
formative model	y_24_ = housing system	compost, cubicle	-	-

## Data Availability

The data presented in this study are available on request from the corresponding author. The data are not publicly available due to their use in official genetic evaluations for dairy cattle.

## References

[1] Fourichon C., Seegers H., Beaudeau F., Verfaille L., Bareille N. (2001). Health-control costs in dairy farming systems in western France. Livest. Prod. Sci..

[2] Schukken Y.H., González R.N., Tikofsky L.L., Schulte H.F., Santisteban C.G., Welcome F.L., Bennett G.J., Zurakowski M.J., Zadoks R.N. (2009). CNS mastitis: Nothing to worry about?. Vet. Microbiol..

[3] Detilleux J., Theron L., Beduin J.-M., Hanzen C. (2012). A structural equation model to evaluate direct and indirect factors associated with a latent measure of mastitis in Belgian dairy herds. Prev. Vet. Med..

[4] Leso L., Barbari M., Lopes M.A., Damasceno F.A., Galama P., Taraba J.L., Kuipers A. (2020). Invited review: Compost-bedded pack barns for dairy cows. J. Dairy Sci..

[5] Barberg A.E., Endres M.I., Salfer J.A., Reneau J.K. (2007). Performance and welfare of dairy cows in an alternative housing system in Minnesota. J. Dairy Sci..

[6] Wagner P., Yin T., Brügemann K., Engel P., Weimann C., Schlez K., König S. (2021). Genome-Wide Associations for Microscopic Differential Somatic Cell Count and Specific Mastitis Pathogens in Holstein Cows in Compost-Bedded Pack and Cubicle Farming Systems. Animals.

[7] Pighetti G.M., Elliott A.A. (2011). Gene polymorphisms: The keys for marker assisted selection and unraveling core regulatory pathways for mastitis resistance. J. Mammary Gland. Biol. Neoplasia.

[8] Malek dos Reis C.B., Barreiro J.R., Mestieri L., Porcionato M.A.d.F., dos Santos M.V. (2013). Effect of somatic cell count and mastitis pathogens on milk composition in Gyr cows. BMC Vet. Res..

[9] França M.M., Del Valle T.A., Campana M., Veronese L.P., Nascimento G., Morais J.P.G. (2017). Agentes causadores de mastite e relações entre a CCS com a produção e com a composição do leite em vacas leiteiras. Arch. Zootec..

[10] Klarmann M. (2008). Methodische Problemfelder der Erfolgsfaktorenforschung: Bestandsaufnahme und Empirische Analysen.

[11] Gana K., Broc G. (2019). Structural Equation Modeling with Lavaan.

[12] Hair J.F., Sarstedt M., Hopkins L., Kuppelwieser V.G. (2014). Partial least squares structural equation modeling (PLS-SEM). Eur. Bus. Rev..

[13] de los Campos G., Gianola D., Heringstad B. (2006). A structural equation model for describing relationships between somatic cell score and milk yield in first-lactation dairy cows. J. Dairy Sci..

[14] Wu X.-L., Heringstad B., Gianola D. (2008). Exploration of lagged relationships between mastitis and milk yield in dairy cows using a Bayesian structural equation Gaussian-threshold model. Genet. Sel. Evol..

[15] Casal J., Learte P., Torre E. (1990). A path model of factors influencing bovine leukemia virus transmission between cattle herds. Prev. Vet. Med..

[16] Detilleux J., Theron L., Duprez J.-N., Reding E., Humblet M.-F., Planchon V., Delfosse C., Bertozzi C., Mainil J., Hanzen C. (2013). Structural equation models to estimate risk of infection and tolerance to bovine mastitis. Genet. Sel. Evol..

[17] Blanco-Penedo I., Ouweltjes W., Ofner-Schröck E., Brügemann K., Emanuelson U. (2020). Symposium review: Animal welfare in free-walk systems in Europe. J. Dairy Sci..

[18] Wagner P., Brügemann K., Yin T., Engel P., Weimann C., Schlez K., König S. (2021). Microscopic differential cell count and specific mastitis pathogens in cow milk from compost-bedded pack barns and cubicle barns. J. Dairy Res..

[19] Deutsche Veterinärmedizinische Gesellschaft (2000). Leitlinien zur Entnahme von Milchproben unter antiseptischen Bedingungen und Leitlinien zur Isolierung und Identifizierung von Mastitiserregern.

[20] Ariznabarreta A., Gonzalo C., San Primitivo F. (2002). Microbiological quality and somatic cell count of ewe milk with special reference to staphylococci. J. Dairy Sci..

[21] Zecconi A., Dell’Orco F., Vairani D., Rizzi N., Cipolla M., Zanini L. (2020). Differential Somatic Cell Count as a Marker for Changes of Milk Composition in Cows with Very Low Somatic Cell Count. Animals.

[22] Schwarz D., Santschi D.E., Durocher J., Lefebvre D.M. (2020). Evaluation of the new differential somatic cell count parameter as a rapid and inexpensive supplementary tool for udder health management through regular milk recording. Prev. Vet. Med..

[23] Kirkeby C., Toft N., Schwarz D., Farre M., Nielsen S.S., Zervens L., Hechinger S., Halasa T. (2020). Differential somatic cell count as an additional indicator for intramammary infections in dairy cows. J. Dairy Sci..

[24] Sarikaya H., Werner-Misof C., Atzkern M., Bruckmaier R.M. (2005). Distribution of leucocyte populations, and milk composition, in milk fractions of healthy quarters in dairy cows. J. Dairy Res..

[25] Pappenheim A. (1912). Zur Blutzellfärbung im klinischen Bluttrockenpräparat und zur histologischen Schnittpräparatfärbung der hämatopoetischen Gewebe nach meinen Methoden. Folia Haematologica..

[26] Purcell S., Neale B., Todd-Brown K., Thomas L., Ferreira M.A.R., Bender D., Maller J., Sklar P., Bakker P.I.W., de Daly M.J. (2007). PLINK: A tool set for whole-genome association and population-based linkage analyses. Am. J. Hum. Genet..

[27] Zerbino D.R., Achuthan P., Akanni W., Amode M.R., Barrell D., Bhai J., Billis K., Cummins C., Gall A., Girón C.G. (2018). Ensembl 2018. Nucleic Acids Res..

[28] Yang J., Benyamin B., McEvoy B.P., Gordon S., Henders A.K., Nyholt D.R., Madden P.A., Heath A.C., Martin N.G., Montgomery G.W. (2010). Common SNPs explain a large proportion of the heritability for human height. Nat. Genet..

[29] Karunarathna C.B., Graham J. (2018). 46th European Mathematical Genetics Meeting (EMGM) 2018, Cagliari, Italy, April 18–20, 2018: Abstracts. Hum. Hered..

[30] Halli K., Vanvanhossou S.F., Bohlouli M., König S., Yin T. (2021). Identification of candidate genes on the basis of SNP by time-lagged heat stress interactions for milk production traits in German Holstein cattle. PLoS ONE.

[31] Wald A. (1943). Tests of statistical hypotheses concerning several parameters when the number of observations is large. Trans. Amer. Math. Soc..

[32] ENSEMBL Genome Browser. http://www.ensembl.org/.

[33] National Center for Biotchnology Information (NCBI). https://www.ncbi.nlm.nih.gov/.

[34] Kanehisa M., Goto S., Sato Y., Kawashima M., Furumichi M., Tanabe M. (2014). Data, information, knowledge and principle: Back to metabolism in KEGG. Nucleic Acids Res..

[35] R Core Team (2019). https://www.r-project.org.

[36] Rosseel Y. (2012). lavaan: An R Package for Structural Equation Modeling. J. Stat. Soft..

[37] Chin W.W. (1998). The partial least squares approach to structural equation modeling. Modern Methods for Business Research.

[38] Beaudeau F., Fourichon C., Seegers H., Bareille N. (2002). Risk of clinical mastitis in dairy herds with a high proportion of low individual milk somatic-cell counts. Prev. Vet. Med..

[39] Paape M.J., Bannerman D.D., Zhao X., Lee J.-W. (2003). The bovine neutrophil: Structure and function in blood and milk. Vet. Res..

[40] Riggio V., Portolano B., Bovenhuis H., Bishop S.C. (2010). Genetic parameters for somatic cell score according to udder infection status in Valle del Belice dairy sheep and impact of imperfect diagnosis of infection. Genet. Sel. Evol..

[41] Dosogne H., Vangroenweghe F., Mehrzad J., Massart-Leën A.M., Burvenich C. (2003). Differential leukocyte count method for bovine low somatic cell count milk. J. Dairy Sci..

[42] Schwarz D., Diesterbeck U.S., König S., Brügemann K., Schlez K., Zschöck M., Wolter W., Czerny C.-P. (2011). Microscopic differential cell counts in milk for the evaluation of inflammatory reactions in clinically healthy and subclinically infected bovine mammary glands. J. Dairy Res..

[43] Sordillo L.M., Streicher K.L. (2002). Mammary gland immunity and mastitis susceptibility. J. Mammary Gland Biol. Neoplasia.

[44] Tenhagen B.-A., Köster G., Wallmann J., Heuwieser W. (2006). Prevalence of mastitis pathogens and their resistance against anti-microbial agents in dairy cows in Brandenburg, Germany. J. Dairy Sci..

[45] Piessens V., van Coillie E., Verbist B., Supré K., Braem G., van Nuffel A., de Vuyst L., Heyndrickx M., de Vliegher S. (2011). Distribution of coagulase-negative Staphylococcus species from milk and environment of dairy cows differs between herds. J. Dairy Sci..

[46] Craig A.-L., Gordon A.W., Hamill G., Ferris C.P. (2022). Milk Composition and Production Efficiency within Feed-To-Yield Systems on Commercial Dairy Farms in Northern Ireland. Animals.

[47] Sørensen L.P., Madsen P., Mark T., Lund M.S. (2009). Genetic parameters for pathogen-specific mastitis resistance in Danish Holstein Cattle. Animal.

[48] Li M., Lu G., Hu J., Shen X., Ju J., Gao Y., Qu L., Xia Y., Chen Y., Bai Y. (2016). EVA1A/TMEM166 Regulates Embryonic Neu-rogenesis by Autophagy. Stem Cell Rep..

[49] Shen X., Kan S., Liu Z., Lu G., Zhang X., Chen Y., Bai Y. (2017). EVA1A inhibits GBM cell proliferation by inducing autophagy and apoptosis. Exp. Cell Res..

[50] Tian W., Yang X., Yang H., Lv M., Sun X., Zhou B. (2021). Exosomal miR-338-3p suppresses non-small-cell lung cancer cells metastasis by inhibiting CHL1 through the MAPK signaling pathway. Cell Death Dis..

[51] Huang X., Zhu L.-l., Zhao T., Wu L.-y., Wu K.-w., Schachner M., Xiao Z.-C., Fan M. (2011). CHL1 negatively regulates the proliferation and neuronal differentiation of neural progenitor cells through activation of the ERK1/2 MAPK pathway. Mol. Cell. Neurosci..

[52] Khan M.Z., Khan A., Xiao J., Ma J., Ma Y., Chen T., Shao D., Cao Z. (2020). Overview of Research Development on the Role of NF-κB Signaling in Mastitis. Animals.

[53] Ivemeyer S., Knierim U., Waiblinger S. (2011). Effect of human-animal relationship and management on udder health in Swiss dairy herds. J. Dairy Sci..

[54] Hussein M.M., El Agawany A.A.A. (2009). Impact of age at first calving on reproduction, lactation, postpartum disorders and longevity in Holsteins under Egyptian circumstances. J. Vet. Med. Res..

[55] Pirlo G., Miglior F., Speroni M. (2000). Effect of age at first calving on production traits and on difference between milk yield returns and rearing costs in Italian Holsteins. J. Dairy Sci..

[56] Eastham N.T., Coates A., Cripps P., Richardson H., Smith R., Oikonomou G. (2018). Associations between age at first calving and subsequent lactation performance in UK Holstein and Holstein-Friesian dairy cows. PLoS ONE.

[57] Sawa A., Siatka K., Krężel-Czopek S. (2019). Effect of Age at First Calving on First Lactation Milk Yield, Lifetime Milk Production and Longevity of Cows. Ann. Anim. Sci..

[58] Drews J., Czycholl I., Junge W., Krieter J. (2018). An evaluation of efficiency in dairy production using structural equation modelling. J. Agric. Sci..

[59] Urban D., Mayerl J., Urban D., Mayerl J. (2014). SEM-Grundlagen. Strukturgleichungsmodellierung: Ein Ratgeber Für Die Praxis.

[60] Gernand E., König S., Kipp C. (2018). Influence of on-farm measurements for heat stress indicators on dairy cow productivity, female fertility, and health. J. Dairy Sci..

[61] König S., Wu X., Gianola D., Heringstad B., Simianer H. (2008). Exploration of relationships between claw disorders and milk yield in Holstein cows via recursive linear and threshold Models. J. Dairy Sci..

[62] Naderi S., Bohlouli M., Yin T., König S. (2018). Genomic breeding values, SNP effects and gene identification for disease traits in cow training sets. Anim. Genet..

